# Comprehensive Metabolic Signature of Renal Dysplasia in Children. A Multiplatform Metabolomics Concept

**DOI:** 10.3389/fmolb.2021.665661

**Published:** 2021-07-29

**Authors:** Szymon Macioszek, Renata Wawrzyniak, Anna Kranz, Marta Kordalewska, Wiktoria Struck-Lewicka, Danuta Dudzik, Margot Biesemans, Michał Maternik, Aleksandra M. Żurowska, Michał J. Markuszewski

**Affiliations:** ^1^ Department of Biopharmaceutics and Pharmacodynamics, Medical University of Gdańsk, Gdańsk, Poland; ^2^ Department of Pediatrics, Nephrology and Hypertension, Medical University of Gdańsk, Gdańsk, Poland; ^3^ Centre for Rare Diseases, Medical University of Gdańsk, Gdańsk, Poland

**Keywords:** renal dysplasia, metabolomics, pediatric nephrology, multiplatform approaches, LC-MS, GC-MS

## Abstract

Renal dysplasia is a severe congenital abnormality of the kidney parenchyma, which is an important cause of end-stage renal failure in childhood and early adulthood. The diagnosis of renal dysplasia relies on prenatal or postnatal ultrasounds as children show no specific clinical symptoms before chronic kidney disease develops. Prompt diagnosis is important in terms of early introduction of nephroprotection therapy and improved long-term prognosis. Metabolomics was applied to study children with renal dysplasia to provide insight into the changes in biochemical pathways underlying its pathology and in search of early indicators for facilitated diagnosis. The studied cohort consisted of 72 children, 39 with dysplastic kidneys and 33 healthy controls. All subjects underwent comprehensive urine metabolic profiling with the use of gas chromatography and liquid chromatography coupled to mass spectrometry, with two complementary separation modes of the latter. Univariate and multivariate statistical calculations identified a total of nineteen metabolites, differentiating the compared cohorts, independent of their estimated glomerular filtration rate. Seven acylcarnitines, xanthine, and glutamine were downregulated in the urine of renal dysplasia patients. Conversely, renal dysplasia was associated with higher urinary levels of dimethylguanosine, threonic acid or glyceric acid. This is the first metabolomic study of subjects with renal dysplasia. The authors define a characteristic urine metabolic signature in children with dysplastic kidneys, irrespective of renal function, linking the condition with altered fatty acid oxidation, amino acid and purine metabolisms.

## Introduction

Renal dysplasia, though classified as a rare disease (birth prevalence: 1/2,300) is one of the major causes of chronic kidney disease in childhood. This congenital abnormality of the kidneys is due to early abnormal kidney development which results in malformation of the normal histologic structure of the kidney with the characteristic presence of embryological tissue in the form of undifferentiated and metaplastic tissues. Due to an accompanying reduction in the number of overall nephrons, renal dysplasia may lead to chronic kidney disease (CKD) and with time progress to end-stage renal disease (ESRD). Renal dysplasia is one of the most frequent underlying pathologies in children requiring renal replacement therapy (13.5%) (www.orpha.net). Its treatment focuses mainly on slowing down the progression of CKD.

Renal dysplasia is usually symptomless before complications of CKD develop. It is diagnosed through prenatal or postnatal radiological screening. Ultrasonography reveals a normal-sized or small kidney with increased echogenicity and either absent or poor corticomedullary differentiation, frequently accompanied by the presence of small cysts. The extent of dysplastic changes is extremely variable and when mild is difficult to visualize. Early diagnosis is hampered by the lack of available biomarkers of abnormal kidney differentiation in the initial period of stable renal function. Prompt diagnosis of renal dysplasia is important in terms of management and long term prognosis due to the risk of future end-stage renal disease.

Metabolomics is an advanced tool providing insight into molecular processes occurring in a living organism and enabling the observation of disturbances in metabolic pathways resulting from changes in both genome and proteome or from environmental factors. In untargeted metabolomics, a whole set of metabolites present in a studied biological matrix is analysed and subsequently evaluated. Due to the wide range of compounds with various physicochemical properties, usually several complementary analytical techniques are used to cover the whole metabolome. Initially, metabolomics was used in search of disease biomarkers that are challenging for traditional diagnosis (López-López et al., 2018; Parfieniuk et al., 2018) and increasingly for elucidating molecular mechanisms of various disorders such as cancers (Armitage and Barbas, 2014; Kaushik and DeBerardinis, 2018), cardiovascular diseases (Rhee and Gerszten, 2012; Ussher et al., 2016) but also renal diseases (Hocher and Adamski, 2017; Kalim and Rhee, 2017). Detailed knowledge about the molecular basis of a disease may be a starting point for the proposition of new therapeutic targets.

The kidney diseases that are most frequently studied by metabolomics include CKD, diabetic nephropathy, renal cell carcinoma, and acute kidney injury (Kalim and Rhee, 2017). Kidney function, assessed by the estimated glomerular filtration rate (eGFR), has been related to about one third of the detected metabolites in both general and CKD populations (Benito et al., 2018). Studies including the pediatric population are scarce since only one study performed on children was found. Atzori et al. (2010) collected a group of children with various nephrouropathies (renal dysplasia, vesico-ureteral reflux, urinary tract infection, acute kidney injury, and others), and compared their ^1^H NMR-based metabolic profile with healthy children. However, only five children with renal dysplasia were employed, and for statistical analysis dysplasia samples were combined with other pathologies.

The aim of the performed study was to search for urine metabolites which may discriminate children with dysplastic kidneys from those with normally developed ones, taking into account the confounding presence of metabolites characteristic for decreased glomerular filtration rate associated with CKD, which is a characteristic hallmark for this disorder.

## Materials and Methods

### Study Design and Population

72 children were enrolled in the study; 39 subjects with renal dysplasia [mean age 5.68 years (range 0.08–17.40)] and 33 healthy controls [mean age 7.28 years (range 0.09–17.69)]. The majority of children in both cohorts were below 5 years of age. Males were predominant in both the study (66.6%) and control groups (60.6%). Renal dysplasia was diagnosed by ultrasonography. Dysplastic changes were present in both kidneys (bilateral renal dysplasia) in the majority of subjects (61.5%). The renal function was assessed based on the eGFR, calculated with the new Schwartz formula. CKD with a decreased renal function (eGFR <60 ml/min/1.73 m^2^) was present in 41% of the studied cohort with dysplastic kidneys. The clinical features of the studied pediatric cohorts are presented in Table 1. The study was approved by the local Bioethics Committee of the Medical University of Gdańsk (NKBBN/499/2016, NKBBN/493/2018).

**TABLE 1:** Clinical features of the studied cohorts of 72 children (39 with renal dysplasia and 33 healthy controls).

	Cohort of subjects with renal dysplasia	Cohort of healthy controls	*p*-value
Total number enrolled	39	33	—
Females (%)	13/39 (33.3)	13/33 (39.4)	0.540
Males (%)	26/39 (66.6)	20/33 (60.6)	0.540
Mean age ± SD	5.68 ± 5.84	7.28 ± 5.62	0.135
Range age in years	(0.08–17.40)	(0.09–17.69)	—
<5 years age (%)	23/39 (58.9)	15/33 (45.5)	0.215
>5 years age (%)	16/39 (41.1)	18/33 (54.5)	0.215
Mean BMI ± SD	17.05 ± 5.12	16.48 ± 2.65	0.560
Range BMI	(7.07–19.46)	(8.89–25)	—
eGFR >60 ml/min/1.73 m^2^ (%)	21/37 (56.8)	—	—
eGFR <60 ml/min/1.73 m^2^ (%)	16/37 (43.2)	—	—
Bilateral dysplasia (%)	25/39 (64.1)	—	—
Unilateral dysplasia (%)	14/39 (35.9)	—	—

### Chemicals and Reagents

The LC-MS grade methanol and acetonitrile were purchased from Fisher Scientific (Loughborough, United Kingdom). The mobile phase additive formic acid was from Chem-Lab (Zedelgem, Belgium). Ammonium formate, pentadecanoic acid, pyridine, urease, methoxyamine hydrochloride, *N*,*O*-Bis(trimethylsilyl)trifluoroacetamide (BSTFA) with 1% trimethylchlorosilane (TMCS) and alkane standard mixture for GC were from Sigma-Aldrich (United States, Switzerland, Germany). The Milli-Q PLUS system (Millipore, Austria) was used to obtain ultrapure water for sample dilution and urease solution preparation.

### Sample Collection and Preparation

First morning urine samples were collected to minimalize the effects of diet but also of circadian rhythm or physical activity. The samples were collected in 1.5 ml Eppendorf tubes and placed immediately at −80°C. The samples were stored frozen until the day of analyses, when they were thawed at room temperature.

### Untargeted GC-MS and LC-MS Metabolomic Analysis

The urine samples were analyzed along with the quality control (QC) and blank samples, using two complementary analytical platforms namely, gas chromatography coupled to triple quadrupole mass spectrometry (GC-QQQ/MS) and liquid chromatography coupled to time-of-flight mass spectrometry (LC-TOF-MS). Additionally, in terms of the LC technique, two complementary separation modes were used: reversed-phase (RP) and hydrophilic interaction chromatography (HILIC), both in positive (RP+, HILIC+) and negative (RP−, HILIC−) ionization modes. In RP, lipids and other nonpolar metabolites can be separated while HILIC is suitable for the separation of polar compounds such as amino acids, nucleosides, sugars, organic acids, or amines. This yielded five analytical batches for each urine sample. The detailed protocols of sample preparation and analysis for both LC-MS and GC-MS are provided in the Supplementary Material.

Agilent 1,200 HPLC system coupled to a 6,224 TOF/MS system (Agilent Technologies, Germany) was used to determine the urine metabolic fingerprints. Reversed-phase separation was achieved using a 2.1 mm × 100 mm, 1.8 μm, Zorbax Extend-C18 column (Agilent Technologies, United States), with mobile phase consisting of 0.1% formic acid in water and 0.1% formic acid in acetonitrile. In HILIC mode, 10 mM ammonium formate water solution and acetonitrile were used to enable separation of polar compounds in a Poroshell 120 HILIC 4.6 x 50 mm, 2.7 µm column (Agilent Technologies, United States).

Complementary GC-MS analysis was conducted on a GCMS-TQ8030 system (Shimadzu, Japan). The chromatographic separation was performed in a Zebron ZB-5MS column (30 m × 0.25 mm, 0.25 μm) with helium as a carrier gas. The scan mode from 50 m*/z* to 600 m*/z* was applied.

### Data Processing and Analysis

The software used for raw data processing included: MassHunter Qualitative Analysis version B.06.00 (Agilent Technologies, Germany), MassHunter DA Reprocessor version B.08.00 (Agilent Technologies, Germany), Mass Profiler Professional (MPP, Agilent Technologies, Germany) and Automated Mass Spectra Detection and Identification System (AMDIS, National Institute of Standards and Technology, United States). Only peaks with intensity higher than 5,000 counts and present in at least 80% of samples in a group of children with dysplasia or healthy controls were retained for further data processing. Raw data were normalized with the use of probabilistic quotient normalization (PQN) to correct for differences in urine dilution between the patients.

For statistical analysis, Matlab 2014a (Mathworks, Natrick, MA, United States), SIMCA 16 (Sartorius Stedim Biotech, Sweden) and Metaboanalyst 4.0 (https://www.metaboanalyst.ca) were employed. First, principal component analysis (PCA) was applied for each dataset to verify whether the QC samples were measured identically, regardless of their position in the analytical run. To examine the differences between the renal dysplasia group and the healthy controls, *t*-test or Mann-Whitney *U* test with multiple testing correction was applied, depending on data distribution and equality of variances. The variables with corrected *p* value ≤0.05 were considered as significantly differentiating the compared groups. The same methodology was used to compare renal dysplasia patients with normal and decreased eGFR to separate the influence of impaired renal function from metabolic changes due to abnormal kidney structure.

A supervised multivariate statistical method - partial least squares discriminant analysis (PLS-DA), was used to analyse the predictive power of the metabolites to identify patients with dysplasia, considering relationships between all metabolites, in contrast to univariate methods. For PLS-DA models built in SIMCA 16, CV-ANOVA values were calculated to assess their reliability. Based on the PLS-DA models, VIP (variable importance in projection) and SR (selectivity ratio) values were calculated to select compounds that are potentially related to the differentiation between the groups. A VIP coefficient higher than one indicates the variables’ relevance for the differentiation between the compared groups. SR, a further tool for ranking variables importance in regression models, was used for the selection of metabolites that have a different abundance in renal dysplasia patients and healthy controls. For correlation analysis of metabolite abundances with the eGFR values of the patients, Spearman’s rank correlation was calculated.

### Metabolite Identification

In the LC-MS analysis, identification of the analytical signals was the last step of the workflow, following the statistical comparisons. The metabolites were annotated using the measured accurate mass and isotopic distribution pattern, while their identity was confirmed after a fragmentation pattern analyses. For the confirmation of metabolite structures, MS/MS analysis on HPLC-Q-TOF/MS System 6550A (Agilent Technologies, Germany) was implemented. Therefore, the metabolite identification was provided at level 2 according to Metabolomics Standards Initiative. For GC-MS data, annotated metabolites were selected based on their retention indices, calculated from the retention times of the alkane mixture. Further identification of the signals was possible by comparing the metabolite fragments in mass spectra libraries, such as NIST 11 and an in-house library of urinary metabolites.

## Results

### Untargeted GC-MS and LC-MS Metabolomic Analysis

**FIGURE 1:**
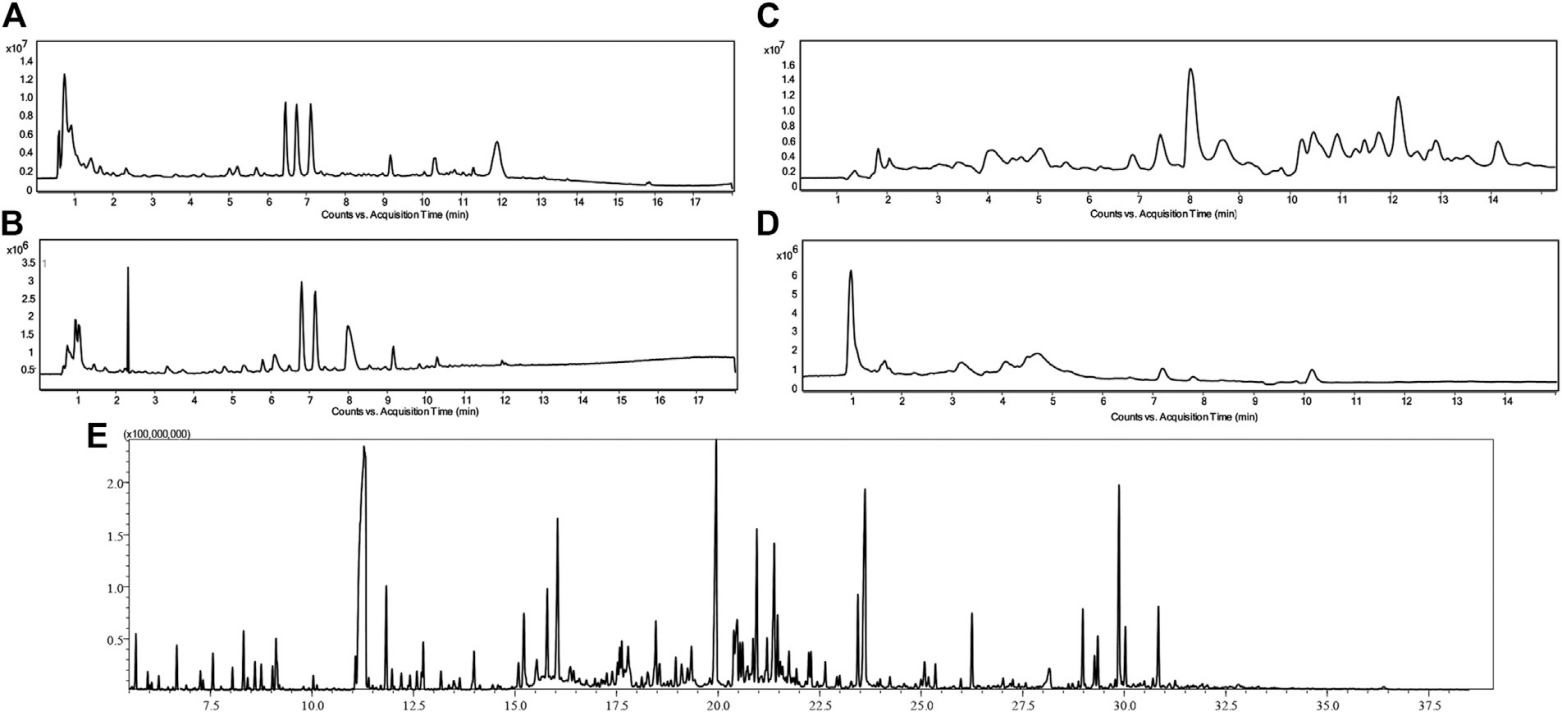
Chromatograms of urine metabolic fingerprints measured by means of RP LC-TOF-MS in positive **(A)** and negative **(B)** ionization modes, HILIC LC-TOF-MS in both positive **(C)** and negative **(D)** ionization modes and GC-QQQ/MS **(E)** in a child with dysplastic kidneys.

Urine metabolic fingerprints from 72 children (39 with renal dysplasia and 33 healthy controls) were measured by means of two complementary analytical platforms, LC-TOF-MS (RP and HILIC in both positive and negative ionization modes) and GC-QQQ/MS. The yielded datasets consisted of 252 (RP+), 54 (RP−), 383 (HILIC+), 32 (HILIC−) and 66 features (GC-MS) after alignment and filtration. Figure 1 illustrates the metabolic fingerprints obtained from the urine of a child with dysplastic kidneys by the two complementary platforms and for LC–MS in the four different separation modes.

**FIGURE 2:**
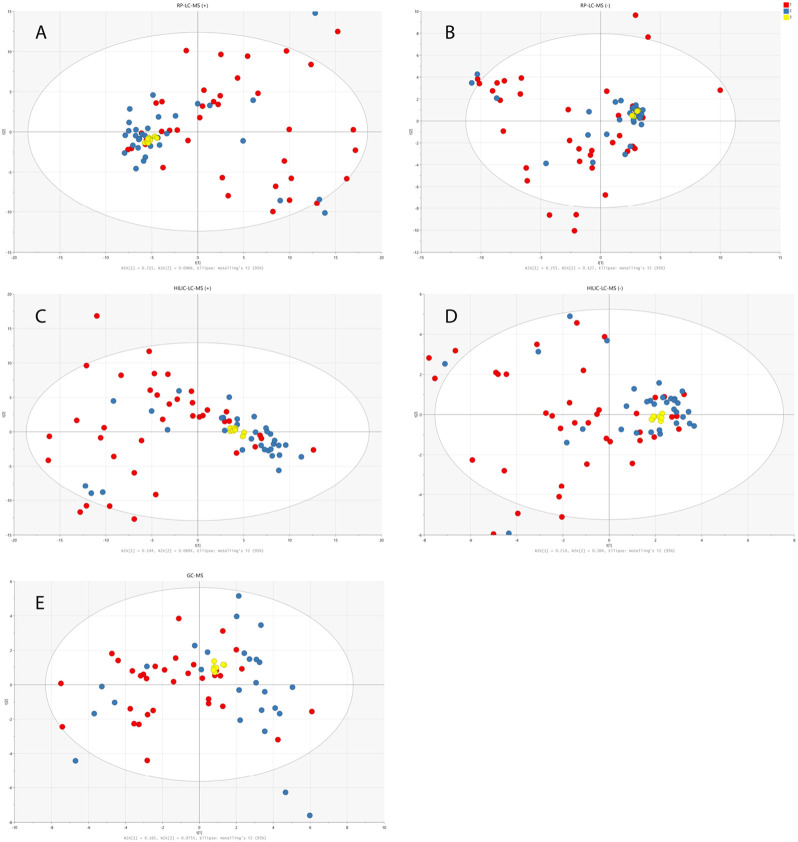
PCA models built on data obtained from RP-LC-TOF-MS analysis in positive **(A)** and negative **(B)** ionization modes, HILIC-LC-TOF-MS analysis in positive **(C)** and negative **(D)** ionization modes and from GC-MS analysis **(E)**. Red circles correspond to samples from disease group, blue ones represent healthy controls and yellow ones QC samples.

PCA models built on PQN-normalized and log-transformed data demonstrated the clustering of the QC samples on the PCA score plots verifying the stability of the analytical system and the method reproducibility. The wide spread of studied urine samples from children with dysplastic kidneys and healthy controls confirmed the validity of the applied experimental procedures and the negligibility of the analytical variability in comparison to the obtained biological variability. The distribution of the samples from both studied cohorts, in comparison to the QC samples, is presented in Figure 2.

### Comparison of Urine Metabolomic Profiles Derived From Children With Renal Dysplasia and Healthy Controls

**FIGURE 3:**
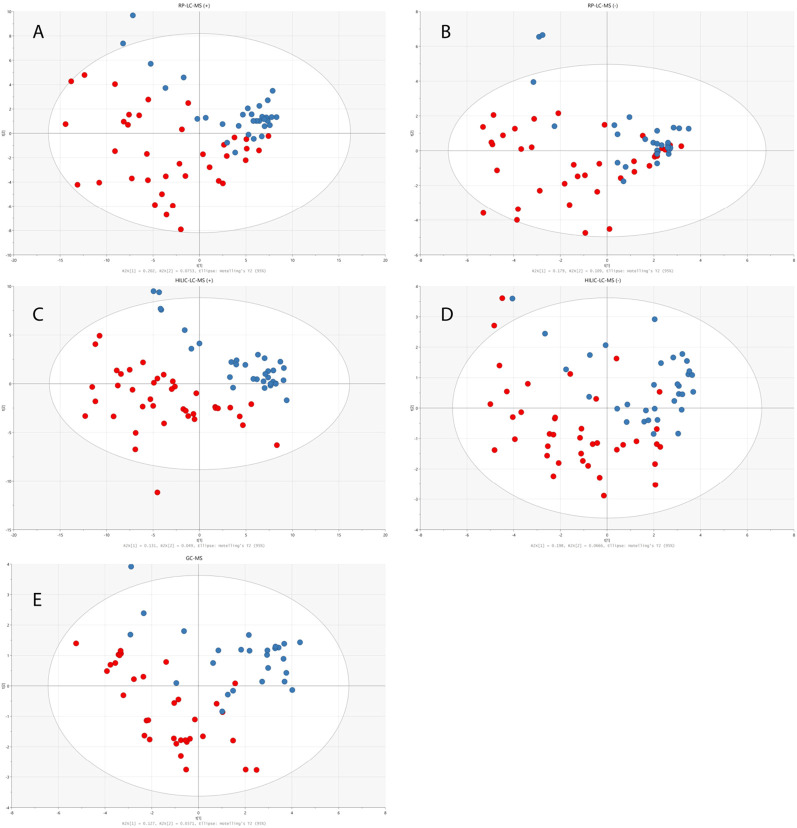
PLS-DA models built on data obtained from RP-LC-TOF-MS analysis in positive **(A)** (*R* ^2^ = 0.25, Q^2^ = 0.282, CV-ANOVA *p* < 0.001) and negative **(B)** (*R* ^2^ = 0.453, Q^2^ = 0.236, CV-ANOVA *p* = 0.013) ionization modes, HILIC-LC-TOF-MS analysis in positive **(C)** (*R* ^2^ = 0.227, Q^2^ = 0.505, CV-ANOVA *p* < 0.001) and negative **(D)** (*R* ^2^ = 0.264, Q^2^ = 0.271, CV-ANOVA *p* < 0.001) ionization modes and from GC-MS analysis **(E)** (*R* ^2^ = 0.164, Q^2^ = 0.174, CV-ANOVA *p* = 0.017). Red circles correspond to samples from disease group, while blue ones represent healthy controls.

The total number of statistically significant variables in the comparison of healthy and disease groups was 44 for RP-LC-MS(+), 10 for RP-LC-MS(−), 52 for HILIC-LC-MS(+), 10 for HILIC-LC-MS(−), and 7 for GC-MS. All of the features detected by LC-MS were subjected to metabolite identification with the use of available databases, and their identity was confirmed by fragmentation patterns analyses with the use of LC-QTOF/MS. Finally, 28 significant metabolites from univariate statistical analysis were successfully identified. Multivariate PLS-DA analysis distinguished 10 relevant metabolites with a VIP value higher than 1, and eight metabolites with an SR value higher than 0.5 that significantly differentiated children with dysplastic kidneys from healthy controls. Figure 3 illustrates a significant separation of urine samples between children with dysplastic kidneys and healthy controls by multivariate PLS-DA analysis. Significant metabolites obtained from all analytical techniques and ionization modes are compiled in Table 2.

**TABLE 2:** Statistically significant metabolites differentiating patients with renal dysplasia from healthy controls.

Compound	Analytical technique	Retention time (min)	Fragmentation pattern	Statistical analysis	Change in dysplasia	Fold change	Biochemical pathway
Methylguanosine	RP (+)	3.1	166.0843	UV	Decrease	−35%	Purine metabolism
6-Keto-decanoylcarnitine	RP (+)	10.1	60.0806, 85.0283, 271.1543	UV	Decrease	−44%	Fatty acid metabolism
Dodecanedioylcarnitine	RP (+)	10.9	60.0806, 85.0284	UV	Decrease	−36%	Fatty acid metabolism
	HILIC(+)	10.5	297.1695	UV		−37%		
Hydroxyisovaleroylcarnitine	RP (+)	1.6	60.0807, 85.0284, 185.0809	UV	Decrease	−31%	Fatty acid metabolism
Hydroxydecanoylcarnitine	RP (+)	10.3	60.0806, 85.0283, 255.1593	UV	Decrease	−55%	Fatty acid metabolism
Citric acid	RP (−)	1.0	111.0088, 85.0295, 87.0088	VIP, SR	Increase	+31%	Citric acid cycle (TCA)
Hippuric acid	RP (−)	6.8	134.0615, 77.0399, 56.0144	VIP	Decrease	−10%	Phenylalanine metabolism
Furoic acid	RP (−)	1.0	67.0190	SR	Increase	+16%	Microbial metabolism
Dimethylguanosine	RP (−)	5.2	178.0736,220.0839, 192.0892	SR	Increase	+18%	Degradation product of tRNA
Betaine	HILIC(+)	11.5	58.0650, 59.0728	UV, VIP, SR	Increase	+80%	Glycine and serine metabolism, methionine metabolism
Nonanoylcarnitine	HILIC(+)	10.2	60.0805, 85.0283, 243.1591	UV	Decrease	−46%	Fatty acidmetabolism
Tiglylcarnitine	HILIC(+)	11.4	60.0807,85.0284	SR	Decrease	−54%	Fatty acid metabolism
Butyrylcarnitine	HILIC(+)	11.7	60.0805, 85.0282, 173.0808	UV	Decrease	−55%	Fatty acidmetabolism
Trimethylamine N-oxide	HILIC(+)	11.9	58.0649, 59.0728	VIP	Decrease	−34%	Microbial metabolism in diverse environments
Carnitine	HILIC(+)	12.7	57.0334, 60.0807, 85.0284, 103.0391	VIP	Increase	+24%	Fatty acid metabolism
Dimethylarginine	HILIC(+)	13.2	70.0650, 88.0868, 116.0706, 158.1288	VIP	Decrease	−42%	L-arginine derivative
Xanthine	HILIC(−)	2.8	80.9652, 108.0206	UV	Decrease	−43%	Purine metabolism
Uric acid	HILIC(−)	4.6	124.0143, 96.0195, 69.0086	VIP, SR	Increase	+228%	Purine metabolism
Indoxyl sulfate	HILIC(−)	1.0	79.9571,80.9557, 132.0452	UV	Decrease	−18%	Tryptophan metabolite
p-Cresol sulfate	HILIC(−)	1.0	107.0501, 79.9572	VIP, SR	Decrease	−40%	Microbial metabolism
Glutamine	HILIC(−)	7.2	127.0513,128.0353, 109.0407, 101.0720	UV	Decrease	−35%	D-Glutamine and D-glutamate metabolism
Hexadecanoic acid	GC	23.0	313.0,117.0,132.0	UV, SR	Increase	+38%	Fatty acid biosynthesis
Threonic acid	GC	15.9	292.0, 205.0, 220.0	UV, VIP	Increase	+20%	Ascorbate and aldarate metabolism
Glyceric acid	GC	12.3	292.0, 189.0, 133.0	UV	Increase	+234%	Glycerolipid metabolism, glycine and serine metabolism
Arabitol	GC	18.5	307.0,217.0,103.0	UV, VIP	Increase	+51%	Pentose and glucuronate interconversions
Lactose	GC	30.1	361.0,204.0,319.0	UV	Increase	+15%	Lactose synthesis, galactose metabolism
Aconitic acid	GC	18.9	375.0,229.0,285.0	UV	Decrease	−30%	Citric acid cycle (TCA)
Lactic acid	GC	7.3	117.0,191.0	UV	Increase	+270%	Glycolysis/Gluconeogenesis

UV- univariate statistical analysis, VIP- variable importance in projection, SR-selectivity ratio.

### Metabolic Changes Potentially Associated With Renal Function due to Reduced eGFR in Patients With Dysplastic Kidneys

In this study, the urinary metabolomic signature of children with renal dysplasia in comparison to the healthy pediatric controls, was evaluated using complementary analytical platforms and advanced statistics. The observed urinary metabolite changes derived mainly from the purine, lipid and amino acid metabolism as well as from glycolysis, the TCA cycle and the urea cycle. Among the 28 metabolites which were significantly different in renal dysplasia subjects in comparison to the healthy controls, nine were found to differentiate subjects with normal and reduced eGFR (Table 3). The highest correlation with eGFR values was calculated for metabolites shown in Table 4. Due to the presence of CKD (eGFR <60 ml/min/m2) in a significant proportion of the studied cohort, further statistical comparisons were conducted in patients with renal dysplasia based on eGFR criteria. The final set of 19 metabolites which significantly differed subjects with renal dysplasia independently of eGFR from healthy controls is listed in Table 5.

**TABLE 3:** Statistically significant metabolites differentiating patients with normal and reduced estimated glomerular filtration rate (eGFR).

Compound	Analytical technique	Statistical analysis	Biochemical pathway
Methylguanosine	RP (+)	UV	Purine metabolism
Citric acid	RP (−)	VIP	Citric acid cycle (TCA)
Hippuric acid	RP (−)	VIP	Phenylalanine metabolism
Betaine	HILIC(+)	VIP, SR	Glycine and serine metabolism, methionine metabolism
Trimethylamine N-oxide	HILIC(+)	VIP	Microbial metabolism in diverse environments
Carnitine	HILIC(+)	VIP	Fatty acid metabolism
Dimethylarginine	HILIC(+)	UV, VIP	L-arginine derivative
Uric acid	HILIC(−)	VIP	Purine metabolism
p-Cresol sulfate	HILIC(−)	VIP, SR	Microbial metabolism

**TABLE 4:** Metabolites with the highest correlation with eGFR value (Spearman’s rank correlation).

Compound	r	Analytical technique
3-Methylglutarylcarnitine	−0.58	RP+
Methyluric acid	−0.44	RP+
	−0.40	RP-
	−0.60	HILIC-
Methylguanosine	0.77	RP+
	0.63	HILIC+
N-Acetylasparagine	−0.68	HILIC+
Guanidinosuccinic acid	−0.48	HILIC+
Dimethylarginine	0.71	HILIC+
Hypoxanthine	0.72	HILIC+
Xanthine	0.47	HILIC-
Ethanolamine	0.63	GC
Uracil	0.58	GC
D-Allose	0.38	GC
Phenylalanine	0.38	GC

**TABLE 5:** Metabolic signature of renal dysplasia, unrelated to eGFR value.

Compound	Change in dysplasia	Biochemical pathway
6-Keto-decanoylcarnitine	Decrease	Fatty acid metabolism
Dodecanedioylcarnitine	Decrease	Fatty acid metabolism
Hydroxyisovaleroylcarnitine	Decrease	Fatty acid metabolism
Hydroxydecanoylcarnitine	Decrease	Fatty acid metabolism
Furoic acid	Increase	Microbial metabolism
Dimethylguanosine	Increase	Degradation product of tRNA
Nonanoylcarnitine	Decrease	Fatty acidmetabolism
Tiglylcarnitine	Decrease	Fatty acid metabolism
Butyrylcarnitine	Decrease	Fatty acidmetabolism
Xanthine	Decrease	Purine metabolism
Indoxyl sulfate	Decrease	Tryptophan metabolite
Glutamine	Decrease	D-Glutamine and D-glutamate metabolism
Hexadecanoic acid	Increase	Fatty acid biosynthesis
Threonic acid	Increase	Ascorbate and aldarate metabolism
Glyceric acid	Increase	Glycerolipid metabolism, glycine and serine metabolism
Arabitol	Increase	Pentose and glucuronate interconversions
Lactose	Increase	Lactose synthesis, galactose metabolism
Aconitic acid	Decrease	Citric acid cycle (TCA)
Lactic acid	Increase	Glycolysis/Gluconeogenesis

Furthermore, no significant differences were observed between male and female patients, according to the applied uni- and multivariate statistical techniques. The age of the subjects did not influence the differences in findings between the cohorts.

## Discussion

Renal dysplasia constitutes a complex and multifaceted disorder characterized by abnormal renal cell differentiation, which leads to the presence of primitive tubules, interstitial fibrosis, renal cysts and cartilage in the renal parenchyma (Phua and Ho, 2016). The most common etiologies of renal dysplasia include both intrinsic defects in the renal parenchyma’s differentiation and functional or structural obstruction of the lower urinary tract (Woolf et al., 2004). Recently, several genetic mutations, mainly associated with Six2, Wnt, Bmp7, and Hnf1β, and copy number variations have been identified in patients with renal dysplasia (Weber et al., 2006; Braun et al., 2016; Verbitsky et al., 2019). Nevertheless, the pathophysiology and underlying molecular mechanisms of renal dysplasia still remain poorly explored and understood in spite of it being one of the most common causes of renal failure in neonates and a leading cause of CKD in childhood.

To the authors’ best knowledge, no publicly available studies have used metabolomics to investigate renal dysplasia, especially in children. Benito et al. (2018) have evaluated the metabolic indicators of CKD in a cohort which included a pediatric population. The results of this study show increased levels of sphingosine-1-phosphate, n-butyrylcarnitine, and cis-4-decenoylcarnitine in plasma from patients with CKD and decreased level of bilirubin. The authors have stressed that kidney function (estimated by the eGFR) is related to about one third of the detected metabolites in both the general and CKD populations (Benito et al., 2018).

**FIGURE 4:**
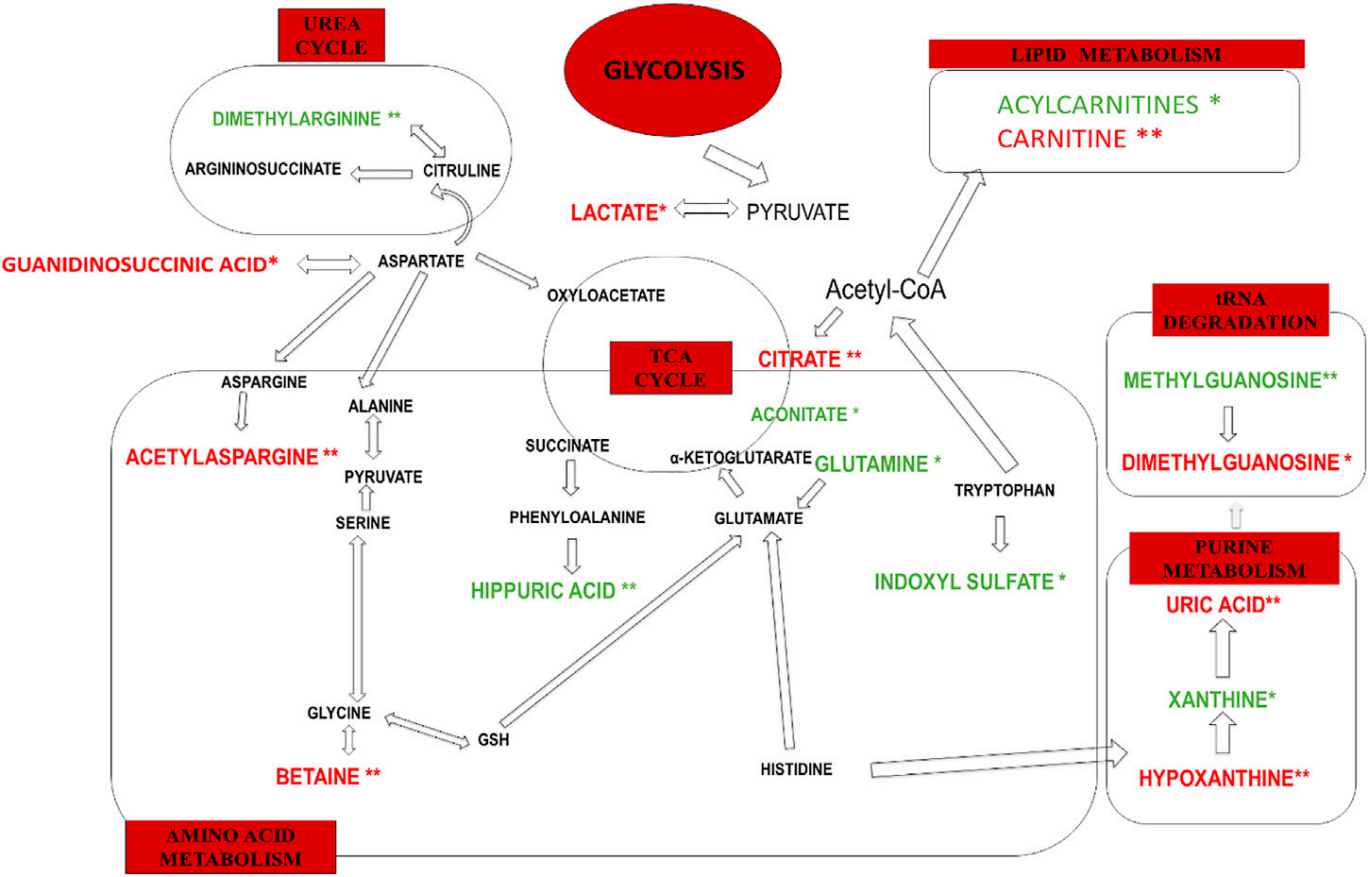
The main biochemical pathways altered only in renal dysplasia (*) and renal dysplasia accompanied by decreased eGFR (**). Decreased levels of urinary metabolites are marked in green. Increased urinary metabolite levels are marked in red.

In this study, the observed urinary metabolite changes derived mainly from purine, lipid and amino acid metabolism as well as from glycolysis, the TCA cycle and the urea cycle. Altered levels of acetylasparagine, trimethylamine-N-Oxide, betaine, dimethylarginine, hippuric acid, uric acid, and hypoxanthine were potentially more characteristic of impaired kidney function measured by decreased eGFR. Most of these metabolites have been previously described in terms of CKD pathophysiology. The main biochemical pathways associated with renal dysplasia and/or a decreased eGFR are graphically displayed in Figure 4.

### Metabolic Changes Related to CKD Pathophysiology

In a recent study based on plasma untargeted metabolomics, the increase in some N-acetyl amino acids was observed in all stages of CKD (*N*-acetylmethionine, *N*-acetylserine, *N*-acetyltryptophan, *N*-acetylglycine, *N*-acetylphenylalanine, *N*-acetylleucine, *N*-acetylproline, *N*-acetyllysine, *N*-acetylasparagine, and *N*-acetylaspartic acid) (Gagnebin et al., 2019). *N-*acetylserine and *N*-acetyllysine were indicated as risk factors of end-stage renal disease for type 1 diabetes and CKD (Niewczas et al., 2017). Sekula et al. (2016) observed that *N-*acetylation might be a crucial detoxification mechanism in CKD. Moreover, *N*-acetylalanine was also observed to be correlated with GFR (Sekula et al., 2016). In the presented study, an increased urinary level of *N*-acetylasparagine was observed in patients with renal dysplasia and was correlated with a decreased eGFR. The above alterations of *N*-acetylated compounds in plasma and urine may indicate a link between *N*-acetylation and renal dysfunction. Trimethylamine-N-oxide (TMAO), a small amine plasma molecule, originates mainly from the intestinal microbiota’s metabolism. Gut microbiota produce trimethylamine (TMA) from food products containing TMA or TMAO and dietary precursors such as choline, phosphatidylcholine, betaine, and carnitine (Al-Waiz et al., 1992; Zhang et al., 1999; Bain et al., 2005). TMA is subsequently absorbed through the intestinal barrier into the bloodstream, then N-oxidized by the hepatic enzyme flavin-containing monooxygenase isoform 3 (FMO3) and excreted as TMAO with urine (de la Huerga et al., 1951; Bell et al., 1991). Thus, TMAO levels could be a result of various production processes, including dietary precursor intake, endogenous TMA production from gut microbiota, TMA and TMAO intestinal absorption, as well as FMO3 enzymatic activity and its renal excretion (Pelletier et al., 2019). Some previous studies have already reported TMAO accumulation in CKD patients (Gagnebin et al., 2019; Pelletier et al., 2019). In this study, a decrease in the urinary level of TMAO was observed in renal dysplasia patients with altered eGFR. A recent study assessed the plasma TMA, TMAO, choline, betaine, and carnitine concentrations in the consecutive stages of CKD (Pelletier et al., 2019) using the measured glomerular filtration rate (mGFR) and the renal clearance. TMAO, choline, and carnitine were inversely correlated with the mGFR in CKD patients.

The elimination of circulating betaine in humans is mainly due to its metabolism rather than renal excretion (Schwahn et al., 2003). However, some previous studies reported that renal excretion of betaine is elevated in patients with kidney injury (Lever et al., 1994; Dellow et al., 1999). In this study the increased urinary level of betaine was observed in renal dysplasia patients with accompanying decreased eGFR, which rather supports the latter opinion. Similar findings were reported by Missailidis at al. (Missailidis et al., 2016), who observed a decrease in the plasma betaine level that was associated with a declined renal function, with the lowest levels observed in stage 5 CKD patients. The results may vary due to different disease stages in these studies. Hippuric acid constitutes the glycine conjugate of benzoic acid, which originates from the phenylalanine metabolism (Zhao et al., 2013). It is primarily eliminated from the blood by the kidneys, through active tubular secretion via organic anion transporters (Deguchi et al., 2005). Additionally, hippuric acid represents one of the well-known protein-bound uremic toxins. In this study, the decreased urinary level of this metabolite was observed as significant in the statistical comparisons between the renal dysplasia patients and the control group, as well as among the renal dysplasia patients with differences in eGFR values. An earlier study also showed the increased tissue level of hippuric acid in CKD rats in comparison to the control group, probably due to the reduced renal clearance of these metabolites. Nevertheless, hippuric acid in humans is also an excretory product of environmental-toxic exposures, dietary protein degradation, and resynthesis by intestinal microbial metabolism of quinic acid through the shikimate pathway (Pero, 2010).

In the presented study, alterations in the purine metabolism were observed in both comparisons, namely in renal dysplasia patients as compared to the control group and in renal dysplasia patients regarding the differences in the eGFR. The metabolism of uric acid is a complex process that includes hepatic production and renal as well as gut excretion (Maiuolo et al., 2016). Uric acid constitutes the end product of both exogenous and endogenous purine metabolisms (Chaudhary et al., 2013). The endogenous production takes place mainly in the liver, intestines, muscles, kidneys, and the vascular endothelium (Chaudhary et al., 2013). Approximately two-thirds of the uric acid load are eliminated by the kidneys, while the remaining one-third is excreted by the gastrointestinal system. Almost all uric acid is filtered from the glomeruli and the amount of its excretion is regulated by post-glomerular reabsorption and secretion (Maiuolo et al., 2016). Reabsorption of uric acid occurs at the S1 segment of the proximal tubule and approximately 10% of the filtered uric acid appears in the urine (Chaudhary et al., 2013). Therefore, hyperuricemia is considered as a crucial risk factor for the renal dysfunction, hypertension, hyperlipidemia, diabetes, and obesity (Maiuolo et al., 2016). Hyperuricemia may be a consequence of the increased production or impaired renal excretion, as well as of a combination of both processes (Su et al., 2014). In this study the increased urinary levels of uric acid and hypoxanthine were observed in renal dysplasia patients as compared to the control group, also in terms of eGFR differences. There are many previous studies that indicated the blood hyperuricemia and disturbed purine nucleotide metabolism as potential contributory risk factors in the development and progression of CKD (Johnson et al., 2013; Mazumder et al., 2018; Zoccali and Mallamaci, 2018; Oh et al., 2019). Thus, the disturbances of the purine metabolism observed in this study, may rather indicate kidney dysfunction than renal dysplasia.

### Metabolomic Signature Potentially Characteristic for Renal Dysplasia

The metabolic changes, observed in this study as statistically significant only between renal dysplasia patients and control group, include decreased urinary levels of acylcarnitines, indoxyl sulfate, xanthine, aconitate, glutamine as well as increased urinary levels of lactate, dimethylguanosine, and guanidinosuccinic acid.

Acylcarnitines, esters of L-carnitine, and fatty acids play a crucial role in the cellular metabolism (Li et al., 2019). The main function of acylcarnitines constitutes long-chain fatty acids (LCFAs) metabolism, as they transport activated LCFAs into the mitochondria for subsequent β-oxidation to provide energy for various cell processes (Tarasenko et al., 2018). Acylcarnitines are also involved in glycolysis, TCA cycle, branched-chain amino acid metabolism, fatty acid peroxidation, and ketone bodies production. Therefore, they are key factors regulating the balance of the intracellular sugar and lipid metabolism (Qu et al., 2016). In this study, the decreased urinary levels of a few acylcarnitines (6-keto-decanoylcarnitine, dodecanedioylcarnitine, hydroxyisovaleroylcarnitine, hydroxydecanoylcarnitine, nonanoylcarnitine, butyrylcarnitine) were observed as statistically significant in renal dysplasia patients as compared to the control group. These alterations can be explained by blood accumulation of acylcarnitines, potentially associated with mitochondrial dysfunction. The matrix of mitochondria constitutes a central place for metabolic pathways such as the TCA cycle and oxidative phosphorylation (OXPHOS) (Podrini et al., 2020). The reduced forms of nicotinamide adenine dinucleotide (NADH) and flavin adenine dinucleotide (FADH_2_) derived from the glycolysis pathway. The fatty acid oxidation (FAO) and TCA cycle are energy-rich molecules containing a pair of electrons with high transfer potential. These electrons are used to reduce molecular oxygen to water and large amount of free energy is released, which subsequently can be used for adenosine triphosphate (ATP) generation. OXPHOS constitutes a process involved in ATP production as a result of the electron transfer from NADH or FADH_2_ to O_2._

**FIGURE 5:**
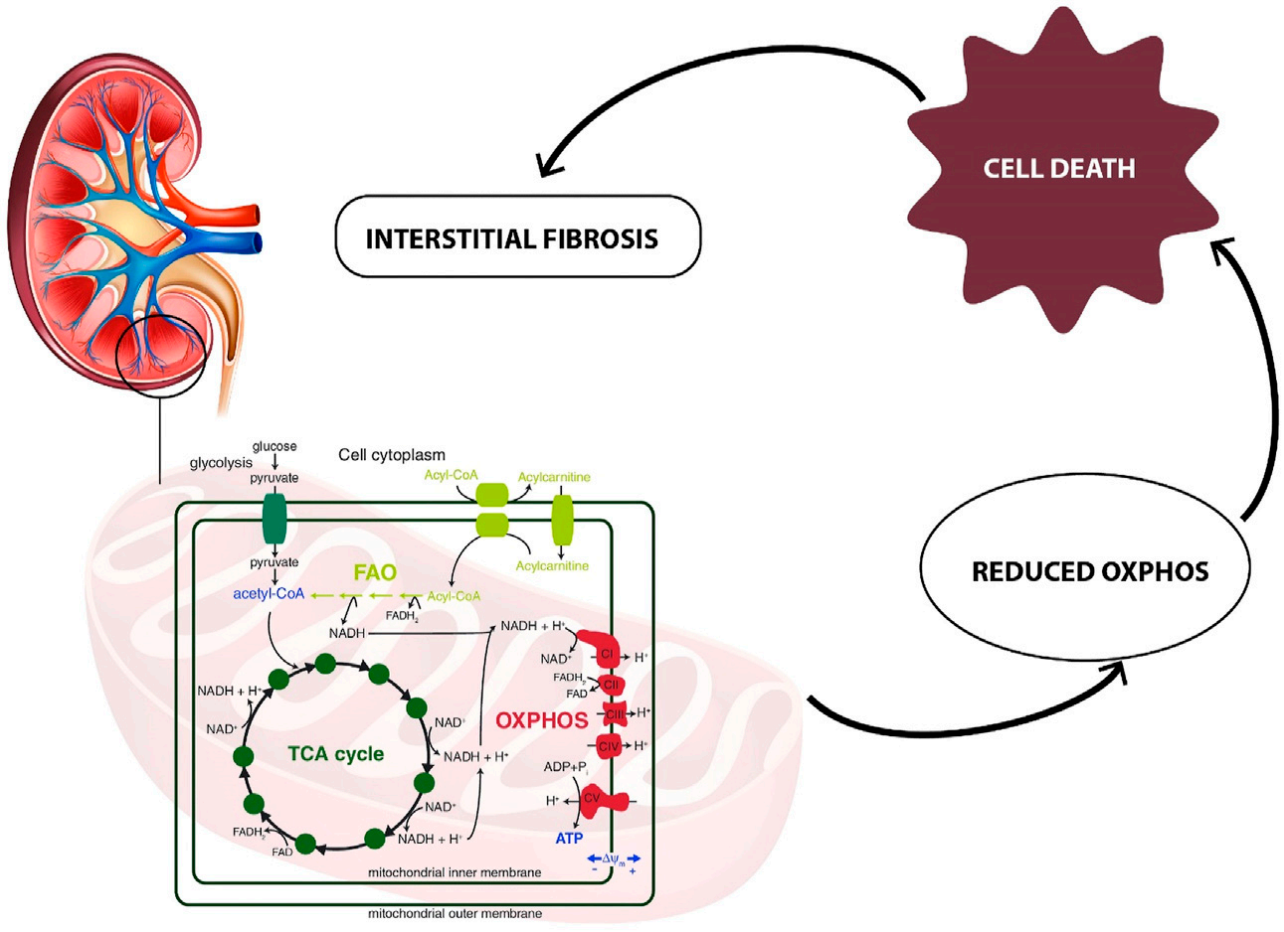
Relationship between metabolic reprogramming of mitochondrial metabolism (FAO, OXPHOS) and kidney interstitial fibrosis.

Several renal diseases, including tubular disorders, chronic tubulointerstitial nephritis, cystic renal disease, and glomerular diseases were reported as mitochondrial cytopathies affecting the OXPHOS activity (Ueda et al., 2004; Au et al., 2007; Emma et al., 2011). One of the recent reports, indicated mitochondrial damage as a key feature of renal inflammation and fibrosis (Chung et al., 2019). In this study, the human kidney tissue and kidney tissue samples collected from animal models with fibrosis were analyzed. The significant mitochondrial defect, including the loss of the mitochondrial transcription factor A (TFAM) in kidney tubular cells, resulting in a reduced OXPHOS, was observed. Additionally, the kidney histological analysis was performed and significant epithelial atrophy, dilated tubules, and interstitial fibrosis were indicated (Chung et al., 2019). Interstitial fibrosis is frequently present in dysplastic kidneys and increases with the progression of CKD. Since FAO is the main energy source for renal proximal tubular epithelial cells, the reduced FAO process would impact the lipid metabolism (Zhou and Liu, 2016). It could lead to disruption of balance between fatty acid synthesis and consumption, as well as dysregulation of intracellular lipid accumulation. Inhibition of FAO in tubular epithelial cells *in vitro* results in ATP depletion, apoptosis, cell dedifferentiation, and intracellular lipid deposition (Kang et al., 2015). The potential relationship between metabolic reprogramming of mitochondrial metabolism (FAO, OXPHOS) and kidney fibrosis is presented in Figure 5.

The metabolic alterations related to the glutamine, aconitate, and lactate levels were also observed to be statistically significant in the comparison between renal dysplasia patients and the control group. All these alterations indicate the involvement of the aerobic glycolysis (a Warburg-like effect), glutamine anaplerosis and the dysregulation of fatty acid biosynthesis. These metabolic changes are also connected with the above described reduced FAO and OXPHOS activities. Recently, the same metabolic reprogramming related to mitochondrial dysfunction has been observed in polycystic kidney disease (Podrini et al., 2020). Additionally, Zhao et al. (2016) reported these metabolic alterations in renal fibrosis in rats, which suggests that the cells utilize mainly the glucose and lipid metabolism to maintain energy homeostasis during renal fibrotic process.

In this report, the decrease in urinary level of indoxyl sulfate was observed in renal dysplasia patients as compared to control group. The gut microbiota convert the dietary tryptophan to indole, which is absorbed by the intestine and subsequently metabolized to indoxyl sulfate in the liver (Pezzatti et al., 2019). Indoxyl sulfate represents another established uremic toxin (Pezzatti et al., 2019). The above-mentioned metabolic alteration observed in this study, may be related to renal dysfunction and altered kidney elimination of indoxyl sulfate. However, in the recent ^1^H-NMR-based kidney and urine untargeted metabolomics of renal interstitial fibrosis rats, the altered level of indoxyl sulfate was observed (Zhao et al., 2016). Zhao et al. (2013) reported the increased level of indoxyl sulfate in the kidney of a rat model of early renal injury, using UPLC Q-TOF/HSMS untargeted metabolomics approach. Moreover, many of previous studies, marked this metabolite as indicator of renal function, vascular disease and mortality in CKD patients (Gika et al., 2012; Gagnebin et al., 2019; Pezzatti et al., 2019). By contrast, in the longitudinal metabolomics studies, plasma levels of indoxyl sulfate showed no association with incident CKD (Rhee et al., 2013) or with CKD progression (Niewczas et al., 2014). The observed results of the above-mentioned studies may be associated with various biological models used, different range of patients’ age or the stage of the disease. However, indoxyl sulfate seems to be a metabolic indicator of early kidney dysfunction or renal histopathological changes.

Xanthine represents a significant metabolic byproduct of guanine triphosphate (GTP) or guanine metabolism and is derived from the purine metabolism pathway (Giulia Battelli et al., 2016). The elevated plasma or urinary xanthine levels may result from the inhibition of xanthine oxidase or from the blockage of the metabolism of xanthine to uric acid (Marro et al., 1997). In this study, the decline in urinary level of xanthine was observed in renal dysplasia patients as compared to the control group. The decrease of this metabolite in the kidneys of rats with renal fibrosis was also recently reported (Zhou and Liu, 2016). This alteration may underline the reduced purine metabolism and bioenergy production. The same trend in xanthine levels in the kidneys was observed in an animal model of early renal injury (Zhao et al., 2013).

Dimethylguanosine, a modified nucleotide indirectly associated with purine metabolism, constitutes a degradation product of transfer RNA and is mainly excreted by the kidneys (Tsalik et al., 2015). In this study, the elevation in urinary level of dimethylguanosine was observed in the renal dysplasia group as compared to the healthy subjects. Previously, consistently higher serum levels of this metabolite were reported in polycystic kidney disease (Grams et al., 2017). Probably, the early decline in kidney blood flow in polycystic kidney disease compared with GFR differentially affects the secretion of small molecules by the proximal tubule. Dimethylguanosine was also indicated to have reduced urinary excretion in patients with kidney failure related to proximal tubule function (Niwa et al., 1998). Additionally, the elevated plasma level of dimethylguanosine was observed in patients with acute renal injury (Tsalik et al., 2015).

Guanidinosuccinic acid (GSA), a derivative of L-arginine, is a precursor of nitric oxide (NO) which tends to accumulate in uremic plasma (Zerra and Josephson, 2019). GSA constitutes also an example of a well-known uremic toxin. GSA impairs the secondary wave of ADP-induced platelet aggregation as well as the release and synthesis of thromboxane A2 in platelets in advanced renal disease (Zerra and Josephson, 2019). In this study, the statistically significant elevation in urinary level of GSA was observed in the renal dysplasia subjects as compared to the control group. Previously, the increased serum level of GSA was reported in end-stage renal failure patients (de Deyn et al., 2003). Additionally, the accumulation of guanidino compounds has been associated with neurological, cardiovascular, hematological, and immunological complications of renal failure (Ringoir et al., 1988). The increased levels of GSA were also reported in the plasma and kidneys of a rat model of polycystic kidney disease (Torremans et al., 2006).

In summary, the urine metabolic changes discovered in children with dysplastic kidneys seem to be characteristic and point towards the presence of altered fatty acid oxidation, amino acid, and purine metabolisms in this parenchymal disorder. However, there are some limitations of this research. The studied renal dysplasia group included mostly young children on different diets and with a significant proportion of subjects with a decreased GFR. Furthermore, while decreased renal function has been reported to influence the metabolic findings in renal diseases, the influence of age and diet requires more extensive investigation. It is also difficult to unequivocally interpret the recognized metabolic signature that is present in children with renal dysplasia as specific for this disorder. It may also represent early abnormalities of the initial stages of CKD which are poorly described. Another option may be that the obtained metabolic signature is due to the presence of renal cysts or ongoing processes of kidney fibrosis.

Integration of the obtained metabolic data with further proteomic, genomic or transcriptomics research may unravel the still poorly understood mechanisms of progression of renal dysplasia in the future. Additionally, it may facilitate earlier recognition of renal dysplasia and enable the introduction of novel therapies for nephroprotective management of children with this congenital abnormality.

## Conclusion

The novel application of a comprehensive metabolomic analysis enabled the recognition of a characteristic urinary metabolic profile for renal dysplasia, allowing the evaluation of different metabolic pathways involved in this disorder. Metabolites associated with the decreased eGFR were excluded to eliminate the influence of decreased kidney function which has been recognized as an important confounding factor. The main biochemical pathways that have been found to be altered in dysplastic kidneys include the glycolysis pathway, the lipid, purine and amino acid metabolism, and the TCA and urea cycles. We suggest that the decreased levels of acylcarnitines in the urine of the renal dysplasia subjects are caused by their accumulation in the blood, due to mitochondrial dysfunction. In consequence, oxidative phosphorylation and fatty acid oxidation may be disturbed, leading to ATP depletion, apoptosis, cell dedifferentiation, and intracellular lipid deposition. A further validation of the reported results is necessary and should be performed in larger populations of children with renal dysplasia, notably in those with a normal renal function.

## Data Availability

The raw data supporting the conclusions of this article will be made available by the authors, without undue reservation.
