# Supplementary material for: Comprehensive Metabolic Signature of Renal Dysplasia in Children. A Multiplatform Metabolomics Concept
Source: Front Mol Biosci. 2021 Jul 29;8:665661. doi: 10.3389/fmolb.2021.665661 (PMC8358436; doi:10.3389/fmolb.2021.665661)
Supplement: Supplementary file 1 [file Table1.DOCX]

Supplementary Material

# Sample preparation and untargeted GC-MS and LC-MS metabolomic analysis

For LC-MS analysis, sample preparation procedure was based on dilution with ultrapure water. After thawing at room temperature, urine samples were vortex-mixed for 1 min and 500 µl of the sample was mixed with 500 µl of ultrapure water. The diluted samples were then centrifuged at 2469 × *g* for 15 minutes and supernatant was filtered through a 0.2 µm nylon filter to amber glass vials dedicated for LC-MS analysis.

For GC-MS metabolic fingerprinting, additional step of compounds derivatization is required. In a glass tube, 200 µl of urine was vortex-mixed with 50 µl of urease solution prepared by dissolving 42.5 mg of urease in 5 ml of water. The mixture was incubated at 37°C for 30 minutes. Then, proteins were precipitated with 800 µl of cold (-80°C ) methanol and 10 µl of pentadecanoic acid methanol solution (1 mg/ml) was added. The internal standard was used to control the reproducibility of the sample preparation procedure. After 5 min of vortex-mixing, samples were centrifuged for 15 min at 2469 × *g* at 4°C. Next, 200 µl of the supernatant was transferred to a GC-MS glass vial and evaporated to dryness at 30°C for 2 hours. Dry residue was dissolved in 30 µl of methoxyamine solution prepared by mixing 75 mg of methoxyamine in 5 ml of pyridine. Following vortex-mixing for 10 min, the mixture was incubated for 16 hours in a dark place at room temperature. Afterwards, 30 µl of 1% TMCS in BSTFA was added and vortex-mixed for 5 min. The last steps included incubation at 70°C for 1 hour, addition of 70 µl of heptane and vortex-mixing for 5 min.

Identical volumes of all urine samples were pooled to obtain quality control (QC) samples. For LC-MS analysis each QC was prepared by applying sample preparation procedure to 500 µl of the pooled urine, whereas for GC-MS, 200 µl of the pooled urine was used for each QC. . During the sequence run, the QC sample was regularly injected after each 8 urine samples.

As regards LC-MS analysis in RP mode, injection volume was 2 µl and flow rate 0.35 ml/min. The column temperature was held at 35°C. The gradient started at 2% of organic phase B (0.1 % formic acid in acetonitrile), going through 20% at 6 min, 45% at 9 min and reaching 98% of organic phase at 14 min. The highest ratio of phase B was held for 4 min. The total time of gradient elution was set at 18 min, and after each run the column was re-equilibrated for 10 min to achieve initial parameters.

For HILIC LC-MS analysis, 2 µl of samples were injected into the column maintained at 25°C. Mobile phase flow rate was set at 0.4 ml/min. The gradient started at 5% of aqueous phase, was increased to 45% at 12 min and maintained in this proportion for 3 min. Each run lasted 15 min and then, the column was re-equilibrated for 7 min. Above-mentioned parameters were equal for positive and negative ionization modes. Regarding MS settings, most parameters were constant in RP and HILIC separation modes. The nebulizer gas temperature was set at 350°C and its flow rate at 11 l/min. The capillary voltage was 3250 V while the nebulizer pressure was 50 psi in RP mode and 45 psi in HILIC mode. Ions present in urine samples were measured in a mass range from 50 *m/z* to 1100 *m/z*.

In GC-MS, splitless injection was applied with injection volume of 1 µl and the injection temperature 250°C. The temperature gradient was run from 60 °C to 320 °C in 38.5 min.

The stability of both LC-MS and GC-MS systems was controlled by injecting QC samples at consistent intervals throughout the analytical run. Also, blank samples were injected before biological samples to check the system’s suitability and possible contamination. Moreover, at the beginning of GC-MS worklist, a mixture of C10-C40 even alkanes was included for calculation of retention indices of the detected compounds and facilitating metabolite identification.

# Data processing and analysis

For deconvolution of LC-MS spectra in positive ionization mode, the following adducts were chosen: H^+^, Na^+^, NH4^+^, and the neutral loss of water. In negative ionization mode, ions could be formed by deprotonation ([M − H]^−^ ), addition of HCOO⁻ as well as loss of water. Analytical signals lower than 200 counts were treated as background noise. In RP-LC data, only compounds eluting between 1 min and 17min were retained while in HILIC data we filtered out analytes eluting in the first 0.5 min and after 14.5 min. Preprocessed data were then aligned, allowing for 0.1 min RT shift and 10 ppm error in measured *m/z* values. Afterwards, signals were filtered based on QC samples and presence in dysplasia patients and healthy control samples according to quality assurance criteria [1].

In terms of data obtained from GC-MS analysis, the workflow started with metabolite annotation in NIST 11 library, possible due to hard, reproducible ionization technique applied in the system (electron ionization) as well as reproducible retention times. Detected metabolites were then aligned in all samples and filtered similarly to LC-MS data to prepare them for statistical analysis. To compensate for urine dilution variation between samples, all datasets were normalized using probabilistic quotient normalization (PQN), which is a robust method for metabolomics data [2].

After processing of the data, the signal drift was assessed based on the injected QC samples. As they are replicate samples, all QCs should result in the same signal intensities. To observe if the signal was stable, we presented the total signal intensity of QC samples in a plot. In our case, no drift or trend was observed in the data from GC-MS or LC-MS analyses, hence, no drift correction algorithm was applied.

[1] Dunn, Warwick B., et al. "Procedures for large-scale metabolic profiling of serum and plasma using gas chromatography and liquid chromatography coupled to mass spectrometry." *Nature protocols* 6.7 (2011): 1060-1083.

[2] Dieterle F, Ross A, Schlotterbeck G, Senn H. Probabilistic quotient normalization as robust method to account for dilution of complex biological mixtures. Application in 1H NMR metabonomics. Anal Chem. 2006 Jul 1;78(13):4281-90. doi: 10.1021/ac051632c.
